# Survival of taylorellae in the environmental amoeba *Acanthamoeba castellanii*

**DOI:** 10.1186/1471-2180-14-69

**Published:** 2014-03-19

**Authors:** Julie Allombert, Anne Vianney, Claire Laugier, Sandrine Petry, Laurent Hébert

**Affiliations:** 1 International Center for Infectiology Research (CIRI) Legionella pathogenesis group, Université de Lyon, Lyon, France; 2 Inserm, U1111, Lyon, France; 3 Ecole Normale Supérieure de Lyon, Lyon, France; 4 Université Lyon 1, Centre International de Recherche en Infectiologie (CIRI), Lyon, France; 5 CNRS, UMR5308, Lyon, France; 6 ANSES, Dozulé Laboratory for Equine Diseases, Bacteriology and Parasitology Unit, 14430 Goustranville, France

**Keywords:** *Taylorella equigenitalis*, *Taylorella asinigenitalis*, Contagious equine metritis, *Acanthamoeba castellanii*, Endosymbiont

## Abstract

**Background:**

*Taylorella equigenitalis* is the causative agent of contagious equine metritis, a sexually-transmitted infection of Equidae characterised in infected mares by abundant mucopurulent vaginal discharge and a variable degree of vaginitis, cervicitis or endometritis, usually resulting in temporary infertility. The second species of the *Taylorella* genus, *Taylorella asinigenitalis*, is considered non-pathogenic, although mares experimentally infected with this bacterium can develop clinical signs of endometritis. To date, little is understood about the basic molecular virulence and persistence mechanisms employed by the *Taylorella* species. To clarify these points, we investigated whether the host-pathogen interaction model *Acanthamoeba castellanii* was a suitable model for studying taylorellae.

**Results:**

We herein demonstrate that both species of the *Taylorella* genus are internalised by a mechanism involving the phagocytic capacity of the amoeba and are able to survive for at least one week inside the amoeba. During this one-week incubation period, taylorellae concentrations remain strikingly constant and no overt toxicity to amoeba cells was observed.

**Conclusions:**

This study provides the first evidence of the capacity of taylorellae to survive in a natural environment other than the mammalian genital tract, and shows that the alternative infection model, *A. castellanii*, constitutes a relevant alternative system to assess host-pathogen interactions of taylorellae. The survival of taylorellae inside the potential environmental reservoir *A. castellanii* brings new insight, fostering a broader understanding of taylorellae biology and its potential natural ecological niche.

## Background

*Taylorella equigenitalis* is a Gram-negative betaproteobacterium of the Alcaligenaceae family. It is the causative agent of Contagious Equine Metritis (CEM), a World Organisation for Animal Health (OIE), notifiable disease. CEM is a highly contagious sexually-transmitted infection in horses that may lead to abundant mucopurulent vaginal discharge and variable degrees of vaginitis, endometritis and cervicitis [1], causing temporary infertility and occasional abortions have been reported [2]. The presence of *T. equigenitalis* in stallions does not cause clinical signs and long-term asymptomatic carrier mares have also been reported [3]. These symptomless carrier animals are generally considered to play a key role in the dissemination of CEM during mating [4]. Unknown prior to its identification in 1977 [5,6], it is generally assumed that the worldwide dissemination of *T. equigenitalis* was the result of the shipment of carrier stallions and mares both within and between countries [2]. As a consequence, many countries implemented strict regulations and disease surveillance, making CEM one of the most regulated equine diseases worldwide [7]. CEM continues to have a major impact on the economy of the equine industry, limiting movement and trade of horses internationally [2].

The second species of taylorellae—*Taylorella asinigenitalis*—was first reported in 2001 following its isolation from the genital tract of two jacks and a mare [8,9]. Although closely related to *T. equigenitalis* phenotypically [8] and in terms of its genomic characteristics [10], *T. asinigenitalis* has never been reported to cause clinical signs of disease under natural conditions, and is thus considered non-pathogenic. It is important to note that despite this apparent lack of pathogenicity, mares experimentally infected with *T. asinigenitalis* can develop clinical signs of metritis and cervicitis [9], and that *T. asinigenitalis* can persist for a long time in donkeys [11]. We therefore consider *T. asinigenitalis* a potential emerging pathogen that needs to be monitored.

To date, the evolutionary histories of the taylorellae remain unclear. Analysis of the genomes of *T. equigenitalis* and *T. asinigenitalis* reveals that both species share a very similar gene repertoire (≈ 85% of the total genes predicted are common to both *Taylorella* species) but surprisingly, little DNA sequence identity [10,12]. The recently-described taylorellae MultiLocus Sequence Typing (MLST) scheme, which reveals the highly clonal dissemination of taylorellae (especially *T. equigenitalis*), combined with the emergence of new STs over time suggest that Equidae could be contaminated by an external source of *Taylorella* originating from an as yet unidentified natural ecological reservoir. Moreover, genome sequence analysis of Alcaligenaceae members suggests that taylorellae diverged by genome reduction from an ancestor which probably had a less specific ecological niche [13] than present day Taylorellae.

Due to the lack of a suitable host model and molecular genetic tools to manipulate taylorellae, the molecular mechanisms involved in the pathogenicity of taylorellae and their host colonisation capacity remain largely unknown. The main information available to date is (i) that *T. equigenitalis* is able to invade and replicate in equine dermal cells [14] and (ii) that several genes identified *in silico* as potentially involved in bacterium-host interactions such as genes coding for secretion systems and for proteins containing eukaryotic domains, are present in the taylorellae genomes [10,12].

Since the discovery that *Legionella pneumophila* can infect and replicate in free-living amoebae [15], there has been an increasing interest in these professional phagocytes which have been used as an alternative host model to study various aspects of host-pathogen interactions and to characterise bacterial virulence mechanisms [16]. Among the bacteria that have evolved to resist destruction by free-living amoebae (hereinafter called ARB for amoeba-resistant bacteria) [16] we can distinguish (i) true symbionts, which cohabit with the amoeba and maintain a stable host-parasite ratio over a specific period and (ii) pathogens able to lyse the amoebae [17]. As a protective environment for ARB, free-living protozoa represent a potential bacterial reservoir and may act as a vector for bacterial dissemination and colonisation of new niches [18].

In this study, we examined the potential of the bactivorous amoeba *A. castellanii* as a host model for *T. equigenitalis* and *T. asinigenitalis*. We assessed (i) the survival capacity of taylorellae in the presence of *A. castellanii*, (ii) the internalisation of taylorellae by *A. castellanii* and (iii) the impact of taylorellae on *Acanthamoeba castellanii* cultures.

## Methods

### Bacterial strains and growth conditions

The bacterial strains used in this study were as follows: *Escherichia coli* strain DH5α (Invitrogen), *L. pneumophila* serogroup 1 strain Lens [19] and the two recently-sequenced strains *T. equigenitalis* MCE9 [20] and *T. asinigenitalis* MCE3 [10]. The axenic *A. castellanii* strain used in this study was derived from an environmental isolate [21]. *Escherichia coli* was grown at 37°C in Luria-Bertani (LB) medium. *Legionella pneumophila* was grown at 30°C either on buffered charcoal yeast extract (BCYE) agar [10 g.L^-1^ ACES (N-(2-Acetamido)-2-aminoethanesulfonic acid); 10 g.L^-1^ Yeast extract; 2 g.L^-1^ Charcoal; 15 g.L^-1^ agar; 0.4 g.L^-1^ L-cystein; 0.25 g.L^-1^ FeNO_3_; pH 6.9] or in BYE liquid medium. *Taylorella equigenitalis* and *T. asinigenitalis* were grown at 37°C in 5% (v/v) CO_2_ in air for 48 h and 72 h respectively on ready-to-use chocolate agar media (AES Chemunex, Combourg, France). *Acanthamoeba castellanii* cells were grown at 30°C on PYG medium [0.75% (w/v) proteose peptone, 0.75% (w/v) yeast extract and 1.5% (w/v) glucose] [22] and split once a week.

### Bacterial survival following *A. castellanii* co-infection

*Acanthamoeba castellanii* cells were infected with *E. coli*, *L. pneumophila*, *T. equigenitalis* or *T. asinigenitalis* at an MOI (multiplicity of infection) of 50. Infections were synchronised by spinning the bacteria (880 × g, 10 min) and extracellular bacteria removed by washing. Extracellular bacteria were quantified by plating the supernatant, while amoeba-associated bacteria were quantified by plating once the amoebae were lysed (Triton X-100 0.04%, 30 min on ice and vigorous pipetting).

### Bacteria uptake assay by trypan blue quenching

*Escherichia coli*, *T. equigenitalis, T. asinigenitalis* and *L. pneumophila* phagocytosis by *A. castellanii* was measured by trypan blue quenching as previously described [23]. Briefly, bacterial suspensions of *T. equigenitalis* or *T. asinigenitalis* prepared from plate-grown organisms, together with overnight cultures of *E. coli* and 3-day cultures of *L. pneumophila,* were labelled with 5-(and 6-) carboxyfluorescein succinimidyl ester (FSE). *Acanthamoeba castellanii* monolayers (5 × 10^5^ cells/well) were infected with 2.5 × 10^7^ fluorescent bacteria (MOI 50) for each species. Phagocytosis inhibitors were obtained from Sigma-Aldrich (St Louis, MO), solubilised in DMSO and used at a concentration of 10 μM for Cytochalasin D (CytoD) and 2 μM for Wortmannin (Wort). After centrifugation (880 × g, 10 min) to initiate cell-bacterium contact, the plates were incubated at 30°C for 30 min. The medium was then replaced by 50 μl per well of trypan blue solution to quench the fluorescence of non-internalised bacteria. After 1 min of incubation, the fluorescence of internalised bacteria was measured on an Infinite M200 Pro (Tecan, Männedorf, Germany) at an excitation level of 485 nm and an emission of 530 nm.

### Cytotoxicity to *A. castellanii*

The number of viable *A. castellanii* cells remaining after infection with *E. coli*, *T. equigenitalis*, *T. asinigenitalis* or *L. pneumophila* were counted as previously described [21]. *Acanthamoeba castellanii* monolayers were infected for each bacterium with an MOI of 50. Cell-bacterium contact was initiated by centrifugation (880 × *g*, 10 min) and the plate was incubated at 37°C in 5% (v/v) CO_2_ in air. At indicated time points, the monolayers were washed four times with protease-yeast (PY) extract medium, and then 100 μl of PY medium containing 10% (vol/vol) of Alamar blue (Invitrogen, Cergy Pontoise, France) was added to tested wells. After a 12-hour incubation, the OD_570_ and OD_600_ values were determined. The relative degrees of amoeba mortality were calculated by the following equation: [1 *­* (mean(OD_570_ − OD_600_)_infected_/mean(OD_570_ − OD_600_)_uninfected_)] × 100.

### Confocal laser scanning observations

*Acanthamoeba castellanii* cells were seeded onto sterile glass coverslips in 6-well plates at 5 × 10^6^ per well in PY medium and allowed to adhere overnight. Monolayers were infected at an MOI of 50 with fluorescein-labelled *T. equigenitalis* or *T. asinigenitalis*. Infections were synchronised by spinning the bacteria (880 × *g*, 10 min) and extracellular bacteria were removed by washing. Following 4 h of incubation at 30°C, cells were fixed with 4% paraformaldehyde (30 min, 4°C), permeabilised with ice-cold methanol (2 min), washed three times and labelled with rhodamine phalloidin. Coverslips were examined with an inverted confocal microscope (Axiovert 200 M; Zeiss, Thornwood, NJ) equipped with a 63X phase-contrast objective lens (Plan Neofluar [Zeiss]; aperture, 1.4, oil).

### Light microscope observations of *A. castellanii* infected monolayers

*Acanthamoeba castellanii* monolayers were infected at an MOI of 50 with *E. coli*, *L. pneumophila, T. equigenitalis* or *T. asinigenitalis*. Cell-bacterium contact was initiated by centrifugation (880 × *g*, 10 min) and incubated at 37°C in 5% (v/v) CO_2_ in air. Monolayers were observed with a Nikon inverted microscope coupled with an Olympus camera (DP120).

### Influence of heat-killed *A. castellanii* and *A. castellanii* culture supernatant on taylorellae growth

Microfiltered (0.22 μm) supernatant of *A. castellanii* cultured in PYG medium for 5 days and heat-killed *A. castellanii* cells (100°C, 30 min) were inoculated with a *T. equigenitalis* or *T. asinigenitalis* strain at an OD_600_ of 0.1, 0.2 and 0.5. These cultures were incubated for 5 days at 37°C, either in 5% (v/v) CO_2_ in air in a static state or aerobically under agitation (200 rpm). Bacterial growth was measured over time by optical density measurement and plate counts.

## Results

### Evolution of taylorellae concentrations in co-culture with *A. castellanii*

**Figure 1:**
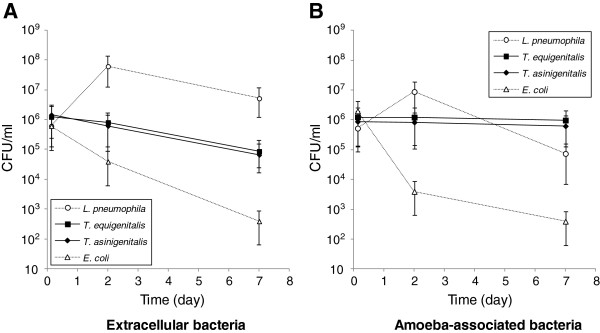
***Taylorella equigenitalis *and *T. asinigenitalis *persist within *A. castellanii *over time.** Evolution of extracellular **(A)** and amoeba-associated **(B)** bacterial concentrations following co-cultures with *A. castellanii* of *T. equigenitalis*, *T. asinigenitalis*, *E. coli* or *L. pneumophila*. Amoebae were infected at an MOI of 50 and at indicated time, extracellular and amoeba-associated bacteria following lysis were quantified by plating. The results are expressed in CFU/ml and each bar represents the geometric mean of triplicate wells. The standard deviations are represented by error bars.

To characterise the capacity of *T. equigenitalis* and *T. asinigenitalis* to persist within the free-living amoeba *A. castellanii*, we performed *A. castellanii*-taylorellae co-cultures and determined the evolution of extracellular (Figure 1A) and amoeba-associated (Figure 1B) bacterial concentrations over time. *Escherichia coli* was used as an amoeba-sensitive control bacterium and *L. pneumophila*, which is able to replicate and evade amoebae, was used as an amoeba-resistant control bacterium. The same evolution of *T. equigenitalis* and *T. asinigenitalis* concentrations was observed over the 7 days of co-culture with *A. castellanii*: the extracellular taylorellae concentrations decreased about one fold over the experiment period, while the amoeba-associated taylorellae concentrations remained strikingly constant throughout. By comparison, the extracellular and amoeba-associated concentrations of *L. pneumophila* rapidly rose after two days of incubation and then declined as expected up to and including day 7, due to the nutrient limitation of the culture medium. As expected, the amount of extracellular and amoeba-associated *E. coli* declined drastically over time during co-culture with *A. castellanii*. These results show that *T. equigenitalis* and *T. asinigenitalis* persist in association with *A. castellanii* over time.

### *Acanthamoeba castellanii*-associated taylorellae are located intracellularly

**Figure 2:**
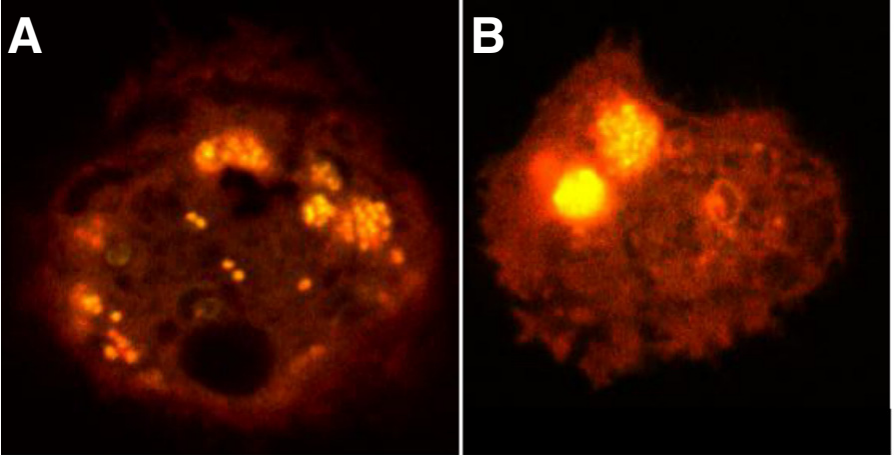
**Location of *T. equigenitalis *and *T. asinigenitalis *within *A. castellanii*.** Confocal laser scanning micrographs of *A. castellanii* cells at 4 h post-infection with fluorescein-labelled *T. equigenitalis***(A)** or *T. asinigenitalis***(B)**. Similar results were observed in two independent experiments.

To determine the location of the amoeba-associated taylorellae, confocal laser scanning micrographs of *A. castellanii* cells were performed four hours after infection with fluorescein-labelled *T. equigenitalis* (Figure 2A) or *T. asinigenitalis* (Figure 2B). For both taylorellae, we observed exclusively intracellular bacteria, mainly grouped in clusters. No bacterium was observed attached to the cell surface of the amoeba. Our data show that the persistent amoeba-associated taylorellae are located within the cytoplasm of *A. castellanii*.

### Actin polymerisation and phosphoinositide 3-kinase play a key role in taylorellae internalisation

**Figure 3:**
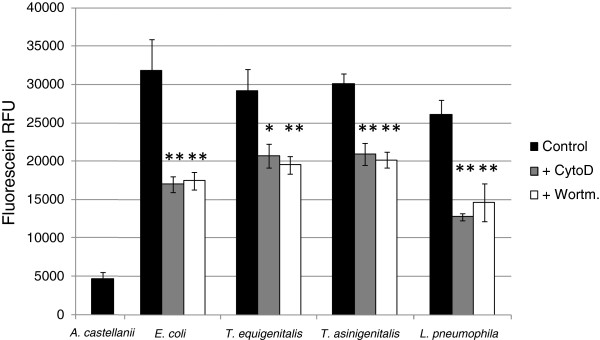
**Taylorellae are actively phagocytised by *A. castellanii*.** Bacterial uptake assay by trypan blue quenching. *Acanthamoeba castellanii* cells were infected with fluorescein-labelled *E. coli*, *T. equigenitalis*, *T. asinigenitalis* or *L. pneumophila* at an MOI of 50, in the presence, when indicated, of either 10 μM of cytochalasin D—an actin polymerization inhibitor (+CytoD)—or 2 μM of Wortmanin—a PI3K inhibitor (+Wort). After 30 min of incubation, the medium was replaced by trypan blue solution to quench the fluorescence of non-internalised bacteria. The fluorescence of internalised bacteria was measured using an excitation level of 485 nm and an emission of 530 nm. Fluorescence data were corrected for differences in labelling efficiency between the tested strains. Each bar represents the mean of triplicate wells and error bars represent the standard deviations. Significant differences from the control, determined by an unpaired tailed Student *t* test, are indicated by *(p = 0.058) and **(p < 0.05).

To investigate the uptake mechanism involved in taylorellae internalisation, two chemical inhibitors were used: Cytochalasin D (CytoD), a potent inhibitor of actin polymerisation, and Wortmannin (Wort), an inhibitor of phosphoinositide 3-kinases (PI3K). Bacterial uptake in amoebae was measured by trypan blue quenching of fluorescein-labelled *T. equigenitalis*, *T. asinigenitalis*, *E. coli* or *L. pneumophila*. Fluorescein-labelled bacteria were used to infect *A. castellanii* when CytoD or Wort were present, as indicated. After contact, trypan blue was added to quench the fluorescence of non-internalised bacteria and the fluorescence, which was representative of bacterial internalisation by amoebae, was measured (Figure 3). For the four tested bacterial species, amoebae exposed to CytoD and Wort show a decrease in fluorescence compared to untreated amoebae. The decrease in fluorescence was comparable for all four bacterial species and for both phagocytosis inhibitors. These results suggest that taylorellae are internalised by an uptake mechanism such as phagocytosis, which is dependent upon actin polymerisation and PI3K.

### Taylorellae do not obviously alter *A. castellanii* physiology

**Figure 4:**
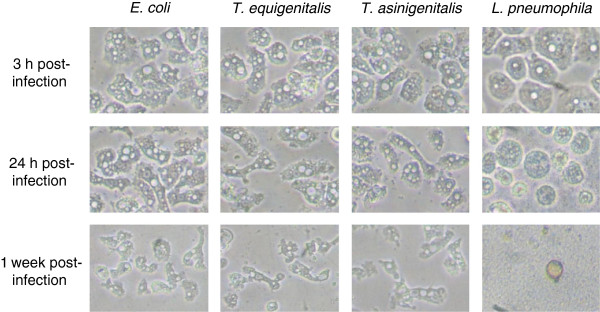
**Evolution of *A. castellanii *monolayers following bacterial infections.** Following infection with *E. coli*, *T. equigenitalis*, *T. asinigenitalis* or *L. pneumophila*, at an MOI of 50, *A. castellanii* monolayers were visualised at an indicated time with an inverted microscope.

In order to visualise the impact of taylorellae on *A. castellanii* physiology, we monitored the evolution of *A. castellanii* morphology over a 7-day incubation period in co-culture with *T. equigenitalis*, *T. asinigenitalis*, *E. coli* or *L. pneumophila* (Figure 4). When *A. castellanii* was cultivated with the amoeba-sensitive *E. coli* bacteria, we observed that the number of amoebae remained stable and that amoeba cells conserved their typical trophozoite appearance, although they became smaller over time probably as a result of the nutrient limitation of the culture medium. In the presence of the amoeba-resistant *L. pneumophila* bacteria, we observed a sharp drop in number of amoeba and a drastic change in the surviving *A. castellanii* cell morphology, which gradually shifted to a stress-induced cyst form. The results obtained for co-cultures with taylorellae were similar to those obtained with *E. coli*, with the observation of a conserved trophozoite appearance, a relatively stable concentration of amoeba and a decrease in the size of amoebic cells. There was no evidence of amoebic cyst formation induced by the presence of *T. equigenitalis* or *T. asinigenitalis*.

**Figure 5:**
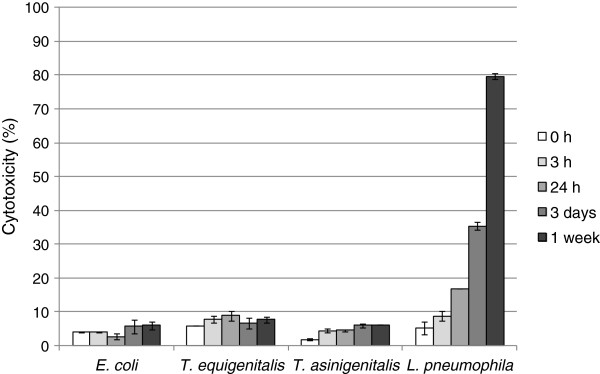
**Taylorellae exhibit low cytotoxicity to *A. castellanii*.***Acanthamoeba castellanii* were infected with *E. coli*, *T. equigenitalis*, *T. asinigenitalis* or *L. pneumophila* with an MOI of 50. The viability of amoebic cells in infected monolayers was quantified at an indicated time using Alamar blue dye. These data are representative of two independent experiments done in triplicate. Each bar represents the mean of triplicate wells; error bars represent the standard deviations.

To assess the toxicity of bacterial species to *A. castellanii*, amoebae were infected at an MOI of 50 with *T. equigenitalis*, *T. asinigenitalis*, *E. coli* or *L. pneumophila*. The viability of amoebic cells in infected monolayers was quantified at indicated time points by using Alamar blue dye (Figure 5). The cytotoxicity of *L. pneumophila* reached 80% after one week of incubation, whereas the cytotoxicity of *T. equigenitalis*, *T. asinigenitalis* and *E. coli* to *A. castellanii* did not exceed 10% after one week. These data reveal that taylorellae have little cytotoxicity effects on *A. castellanii*.

### Taylorellae are not able to grow on dead *A. castellanii* cells

To determine the conditions which allowed taylorellae to persist in the presence of amoebae, we measured *T. equigenitalis* and *T. asinigenitalis* concentrations following 48 h incubation in the presence of either i) PYG medium, ii) PYG medium in which amoebae were previously cultured for 5 days and iii) heat-killed amoebic cells. No taylorellae growth was observed under any of these conditions (data not shown).

## Discussion

Free-living amoebae are ubiquitous predators that control microbial communities and that have been isolated from various natural sources such as freshwater, soil and air [24]. Following studies on the interaction between ARB pathogens (including *Legionella* and *Chlamydia*) and free-living amoebae, it has been suggested that ARB may use free-living amoebae as “training grounds” for the selection of mechanisms of cellular immune evasion [24,25]. In this study, we investigated the interaction of *T. equigenitalis* and *T. asinigenitalis* with the free-living amoeba, *A. castellanii* and showed that taylorellae are able to resist the microbicidal mechanisms of amoebae for a period of at least one week (Figure 1), therefore showing for the first time that taylorellae can be classified as an ARB [16]. However, our results have shown that taylorellae do not induce amoebic death (Figure 4) or cytotoxicity (Figure 5) and indicate that taylorellae are not likely to be considered as amoeba-killing organisms [16].

Confocal microscopic observations of the *A. castellanii*-taylorellae co-cultures also showed that *T. equigenitalis* and *T. asinigenitalis* are found within the cytoplasm of the amoeba (Figure 2), which indicates that taylorellae do not only evade amoebic phagocytosis, but actually persist inside the cytoplasm of this bactivorous amoeba. Moreover, the fact that the phagocytosis inhibitors Wortmannin and Cytochalasin D decrease taylorellae uptake by *A. castellanii* (Figure 3) reveals that actin polymerisation and PI3K are involved in taylorellae uptake*.* This suggests that the internalisation of taylorellae does not result from a specific active mechanism of entry driven by taylorellae, but rather relies on a mechanism involving the phagocytic capacity of the amoeba itself. More investigation on this subject is required to determine the precise effect of taylorellae on organelle trafficking inside the amoeba.

Despite the observed persistence of taylorellae inside amoebae, our results do not allow us to determine whether taylorellae are able to replicate inside an amoeba. During the 7 d of the *A. castellanii*-taylorellae co-cultures, we observed a strikingly constant concentration of *T. equigenitalis* and *T. asinigenitalis*. This phenomenon may be explained either by the existence of a balance between taylorellae multiplication and the bactericidal effect of the amoeba, or by a concurrent lack of taylorellae multiplication and bactericidal effect of the amoeba. Bacterial clusters observed inside *A. castellanii* could be consistent with taylorellae replication within the amoeba, but given that these photographs were taken only 4 h after the co-infection, it seems unlikely that the clusters were the result of intra-amoebic multiplication of taylorellae. Moreover, we observed similar fluorescence levels between taylorellae, whereas the progressive halving of fluorescence within daughter cells following each cell division would have generated differing fluorescence levels between bacteria if bacterial replication had occurred. It appears that this clustering phenomenon is more likely due to the presence of aggregated taylorellae prior to entry into *A. castellanii* or to a trafficking route inside the amoeba that causes gathering of taylorellae at a single location.

In this context, assuming that taylorellae are able to replicate inside amoebae, we can conclude that this phenomenon remains limited and is probably tightly regulated by taylorellae. In order to preserve the protective niche represented by the host cell for as long a duration as possible, it is important that the bacteria do not consume too many nutrients at the detriment of host survival [26]. This statement is consistent with both the limited number of carbon sources which are able to be metabolised by taylorellae [10] and with the absence of observed taylorellae growth in the presence of dead amoebae. Metabolic regulation could be involved in the asymptomatic persistence over several years of taylorellae observed in Equidae [2,27], during which taylorellae could be concealed inside host cells as suggested by the observation of equine dermal cells invasion by *T. equigenitalis*[14]. In this regard, the fact that taylorellae do not induce lysis and that a stable host-parasite ratio remains constant over time, both suggest that taylorellae could be considered a true amoebic endosymbiont, historically defined by Büchner in 1953 as “a regulated, harmonious cohabitation of two nonrelated partners, in which one of them lives in the body of the other” [28].

As highlighted by other intracellular pathogens, protozoan hosts are now considered potential reservoirs and vectors for dissemination of pathogens to mammalian hosts. To date, the natural reservoir of taylorellae is still unknown and it is generally assumed that taylorellae have a limited capacity for survival outside the equine genital tract [29]. In this context, the survival of *T. equigenitalis* and *T. asinigenitalis* in free-living amoebae indicates that protozoa may serve as an environmental reservoir for taylorellae*.* The fact that this capacity is shared by both species of the *Taylorella* genus also suggests that this capacity may have been inherited from a common ancestor. It will therefore be important to broaden our comprehension of taylorellae biology to determine the role played by free-living amoebae in the persistence and dispersal of taylorellae in the environment and to determine, for example, if taylorellae could persist within amoebae during encystment and survive exposure to harsh conditions due to the protection afforded by its amoebic host. We believe that the increasing number of metagenome sequence data sets derived from various environments offer promising ways of identifying taylorellae or taylorellae-related bacteria that will contribute to a clearer understanding of taylorellae biology and to novel insights into the evolution of these microorganisms in the near future. Moreover, it appears interesting in this perspective to establish a parallel between taylorellae and the obligate intracellular chlamydiae that were long recognised only as a phylogenetically distinct, small group of closely related microorganisms before the finding that they were symbionts of free-living amoebae and other eukaryotic hosts, leading to a radical change in the perception of chlamydial diversity [30].

Lateral gene transfer (LGT) is considered a key process in the genome evolution of amoebae and amoeba-associated bacteria. The recent analysis of genes predicted to be derived from LGT in the genome of *Acanthamoeba sp*. [31] showed the presence of 28 genes potentially originating from Betaproteobacteria. Although this analysis did not reveal the presence of genes potentially from taylorellae in *Acanthamoeba*, these results underline the historical relatedness between free-living amoebae and Betaproteobacteria whose different members have been described as naturally infecting free-living amoebae [16,32,33]. On the other hand, no amoeba-related genes were identified during the analysis of taylorellae genomes [10,12]. This observation seems coherent with the plausible evolutionary path of taylorellae reported by Gosh et al., [13] which suggests that the evolution of the taylorellae genome is mainly based on a reduction in size, with very few new gene acquisitions since taylorellae’s separation from the last Alcaligenaceae common ancestor [13].

The capacity of taylorellae to invade and persist inside amoebae supports the usefulness of this inexpensive and easy-to-manipulate host model to assess various aspects of host-pathogen interactions and to characterise the bacterial persistence mechanisms of taylorellae. However, it should be noted that both *T. equigenitalis* and *T. asinigenitalis* behaved in exactly the same way in relation to *A. castellanii*. It is therefore unlikely that all of the variations in virulence level observed in Equidae may be identified. Now that this model has been described, the main limitation to date when studying taylorellae host-pathogen interactions remains the absence of tools needed to genetically manipulate the taylorellae.

## Conclusion

In this study, we investigated the interaction of *T. equigenitalis* and *T. asinigenitalis* with the free-living amoeba, *A. castellanii*. Taken together, our results show that both taylorellae are able to survive for a period of at least one week in amoebic vacuoles without causing overt toxicity to amoeba cells. The *A. castellanii*–taylorellae co-cultures could therefore be used as a simple and rapid model to assess host-pathogen interactions and to characterise taylorellae bacterial persistence mechanisms. Furthermore, this study provides the first evidence of the capacity of taylorellae to survive in a natural environment other than the mammalian genital tract. A full description of this capacity to interact with another eukaryotic host will undoubtedly contribute to a clearer understanding of taylorellae biology and provide new insight into the evolution of these microorganisms.

## Competing interests

The authors declare that they have no competing interests.

## Authors’ contributions

JA Performed and designed the experiments and analyzed the data. JA, AV and LH conceived the study. SP and CL participated in the design of the study and helped to draft the manuscript. LH wrote the paper. All authors read and approved the final manuscript.
